# Treating pregnant and postnatal women with severe mental illness and their infants on a specialised inpatient unit during a pandemic: What are the challenges and lessons learnt?

**DOI:** 10.1192/j.eurpsy.2021.168

**Published:** 2021-08-13

**Authors:** A.-L. Sutter-Dallay

**Affiliations:** Univ. Bordeaux, Inserm, Bph, U1219, F-33000 Bordeaux, France And Perinatal Psychiatry Network-university Department Of Child And Adolescent Psychiatry, Bordeaux University and Charles Perrens Hospital, Bordeaux Cedex, France

**Keywords:** pandemic, joint cares of mothers and babies, Perinatal Psychiatry, Support

## Abstract

From the beginning of the pandemic, pregnant or postpartum women were considered particularly vulnerable. In France, the vast majority of joint care for parents and infants facilities have seen their services closed or the number of people cared for greatly reduced to allow for social distancing to be respected. This notion of social distancing is the antithesis of joint care work, the main objective of which is to support and care for the parent-infant bond by favoring social links Services have had to take ownership of this new situation within a few days and develop new approaches, inventing ways of supporting and linking up at a distance. This presentation will deal in detail with these changes and the solutions proposed, especially kind of home hospitalisations based on discussion groups, the development of programmes to support remote interactions, and also the development of work with fathers, who have been much more present than they usually are, due to the generalisation of teleworking.

**Disclosure:**

No significant relationships.

